# (DIADEM) Digital Assessment of Auditory Perception in Dementia : Study Protocol

**DOI:** 10.1002/alz70856_098358

**Published:** 2025-12-24

**Authors:** Faiza G Durrani, Charles R Marshall, Sheena Waters, Lauren G Bishop, Dicky G Lim, Laura J Smith, Natalia Chemas, Ijeoma Uchegbu, Thamanna G Begum, Alastair J Noyce, Roberta McKee‐Jackson, Ashvini Keshavan, Jason D Warren, Chris JD Hardy, Nicholas Bass

**Affiliations:** ^1^ Queen Mary University of London, London, United Kingdom; ^2^ Queen Mary University of London, London, Greater London, United Kingdom; ^3^ Queen Mary University of London, London, England, United Kingdom; ^4^ UCL, London, United Kingdom; ^5^ Dementia Research Centre, UCL Queen Square Institute of Neurology, University College London, London, United Kingdom; ^6^ Dementia Research Centre, UCL Queen Square Institute of Neurology, London, London, United Kingdom; ^7^ University College London, London, United Kingdom

## Abstract

**Background:**

In Alzheimer's disease (AD), disruption of neural circuits in temporoparietal cortical areas leads to early auditory dysfunction, such as impaired speech‐in‐noise perception. Research shows that poorer speech‐in‐noise performance is associated with lower cognitive performance and reduced grey matter in areas vulnerable to AD. This suggests that auditory cognition could offer a cost‐effective tool for early brain health assessments and dementia risk stratification. Here we describe the protocol for a prospective cohort study evaluating the utility of assessing auditory perceptual function alongside blood‐based biomarkers in a diverse population of people attending memory clinics for evaluation of cognitive symptoms. The DIADEM (Digital Assessment of Auditory Perception in Dementia) study aims to create a scalable, digital test for assessing central auditory processing as an early dementia diagnostic tool. Proof‐of‐concept work showed high accuracy (AUC 0.91) in distinguishing Alzheimer's disease (AD) patients from healthy controls and tracking disease progression. The study will recruit 600 participants from 17 NHS memory clinics in England, including 100 healthy controls, and involve a range of cognitive and auditory tests. Over two years, the study will assess how auditory perceptual function predicts clinical diagnosis, cognitive decline, and dementia progression, particularly in mild cognitive disorder (MCD) patients.

**Method:**

600 participants will be recruited from 17 NHS memory clinics in England.

Participants will undergo speech‐in‐noise, degraded speech, and dichotic sound tests, pure tone audiometry, cognitive assessments (CDR, RUDAS/MMSE), blood biomarkers (pTau‐217, NfL), and MRI.

Participants will be followed for two years with periodic cognitive assessments, CDR, and auditory function evaluations.

**Result:**

We will assess the utility of auditory perceptual tests, both alone and combined with blood‐based biomarkers (pTau217 and NfL), for diagnosis and prognosis in individuals with cognitive symptoms. Additionally, we will examine the cultural fairness and cost‐effectiveness of this approach

**Conclusion:**

This research aims to revolutionize early dementia diagnosis by developing scalable, culture‐fair and precise digital tools based on the measurement of auditory cognition. We aim to improve access to timely and equitable dementia diagnosis and support early treatment and care interventions.

